# Influence of Family Dynamics on Stigma Experienced by Patients With Schizophrenia: Mediating Effect of Quality of Life

**DOI:** 10.3389/fpsyt.2021.645075

**Published:** 2021-08-17

**Authors:** Ling Wang, Yanhua Chen, Chengping Hu, Hongyun Qin

**Affiliations:** Department of Psychiatry, Shanghai Pudong New Area Mental Health Center, Tongji University School of Medicine, Shanghai, China

**Keywords:** family dynamics, quality of life, stigma, schizophrenia, mediating effect

## Abstract

**Background:** Stigma is a barrier to schizophrenia recovery; thus, screening the factors that affect stigma experienced by patients with schizophrenia and exploring the interactions between each factor are critical for improved treatment. The purpose of this study was to demonstrate the relationship between quality of life (QoL), family dynamics, and stigma in patients with schizophrenia.

**Methods:** A total of 447 participants with schizophrenia were recruited in the study, namely, 281 community patients and 166 inpatients. Three scales, Schizophrenia Quality of Life Scale (SQLS), Self-rating Scale of Systemic Family (SSFD), and Stigma Scale for Mental Illness (SSMI), were, respectively, used to evaluate three variables: QoL, family dynamics, and stigma. The correlations between each factor in these three scales were evaluated by Spearman's rank correlation analysis. A mediation model was constructed to investigate whether QoL mediated the relationship between stigma and family dynamics.

**Results:** Correlation analysis revealed that most variables in these three scales correlated significantly with each other. Mediational regression analyses indicated that the degree of stigma was affected by family dynamics; that is, good family dynamics predicted less stigma. Surprisingly, we found that a worse QoL was associated with less stigma, and this led to good family dynamics being related to a worse QoL. These findings further suggested that QoL had a mediating effect on the relationship between family dynamics and stigma.

**Conclusions:** This study suggested that more attention should be focused on the multifactorial influence of stigma on patients with schizophrenia. Integrated and personalized interventions regarding QoL and family dynamics can be tailored for patients with schizophrenia to reduce self-stigma.

## Introduction

Schizophrenia is one of the most severe mental diseases with a lifetime prevalence of about 1%, and approximately two-thirds of new cases of schizophrenia occur before the age of 45 (1). There are over 21 million people living with schizophrenia worldwide, and the number of patients continues to increase (2). Stigmatizing attitudes toward people with mental illness is common and places a burden on stigmatized individuals (3). People with schizophrenia and other mental diseases are often prejudiced by the public, and they may therefore internalize negative attitudes toward their own group, often leading to self-stigma (4). Self-stigma is usually associated with poor quality of life (QoL) and can cause great suffering to patients with schizophrenia (5). Evidence has shown that the degree of self-stigma affects the rehabilitation outcomes of schizophrenia patients (6, 7). In addition, in patients with severe schizophrenia, self-stigma may be the cause and result of adverse outcomes—thus, it may be a potential therapeutic target (8). Therefore, it is necessary to explore the candidate factors that affect the self-stigma in patients with schizophrenia.

Previous studies have emphasized that self-stigma is associated with impaired QoL, and stigma-oriented cognitive and behavioral interventions are needed to improve the QoL of schizophrenia subjects (9). Evidence has also revealed that family support plays an important role in the life of individuals with schizophrenia. Previous studies have indicated that people with schizophrenia are negatively affected when living in a family environment characterized by high levels of criticism, while living in an environment of encouragement and praise by family members can improve the life satisfaction of schizophrenia patients (10, 11). In addition, family dynamics refers to the ways in which family members are connected, so the attitudes and attributions of family members toward individuals with diseases affect the occurrence and development of mental illness (12). Therefore, family intervention for schizophrenia patients can prevent relapse and hospitalizations, and maintain satisfactory family interaction, thereby positively affecting the QoL of patients and relatives (13). Altogether, the factors influencing stigma experienced by schizophrenia patients are complex, and the related interventional treatments are still in an early phase. Although most studies have determined the association between stigma and QoL or the relationship between family dynamics and QoL in patients with schizophrenia, few studies have examined the mediating role of these factors.

Therefore, to reduce the stigma of patients and improve the QoL of patients, further exploration of stigma and its potential factors, such as family dynamics, is needed (14). In this study, we aimed to explore the relationships between stigma, family dynamics, and QoL in patients with schizophrenia. Moreover, a model was constructed to assess whether QoL mediated the relationship between stigma and family dynamics. We hypothesized that (1) higher QoL was connected with a lower level of stigma, (2) good family dynamics would be associated with a lower level of experienced stigma, and (3) QoL might act as a mediating variable between family dynamics and stigma.

## Materials and Methods

### Study Design and Data Collection

A cross-sectional survey was conducted with schizophrenia patients in Shanghai, using self-reporting scales. Inclusion criteria of participants were as follows: patients diagnosed with schizophrenia according to the International Classification of Diseases (ICD-10), patients in a stable phase, patients with a Positive and Negative Syndrome Scale (PANSS) <50 who were assessed by a psychiatrist, patients over 18 years old, and those with a junior high school education and above. Exclusion criteria included major physical illness experienced by patients or family numbers, inability to complete scales because of cognitive dysfunction or communication difficulties, and a high suicidal ideation. In addition, the self-report could reflect the participants' status, beliefs, and perceptions.

Aligned with the inclusion and exclusion criteria, patients from six community health service centers in Pudong New Area (Shanghai), as well as from the Shanghai Pudong New Area Mental Health Center, were screened. Subsequently, we distributed 300 questionnaires to eligible inpatients and community patients. Participants completed the questionnaires on a mobile terminal. Staff in the study team received unified training before the investigation and helped to explain questions that participants might not understand. Questionnaires with a response rate of <50% or with inconsistent results in the same item type due to patient's random answers were excluded. Ultimately, 447 sets of valid data were collected, including data from 281 community patients and 166 inpatients, indicating that the effective response rate of the community was 93.6% and hospital 55.3%. The results showed that the return rate of valid questionnaires in the hospital was lower than in the community. Thus, we randomly interviewed a hospitalized patient, and this patient answered, “I don't want to participate, it is meaningless.” Previous studies have revealed that social isolation during hospitalization can cause a series of long-lasting pathophysiological characteristics, leading to disorders related to schizophrenia, such as social withdrawal (15). This finding may be used to explain the imbalance between community and hospital patients in this analysis.

This study was approved by the Ethics Committee of Shanghai Pudong Mental Health Center (No. 201840372). All patients signed an informed consent form after the procedures and purpose were explained.

### Measures

Demographic information, including gender, age of disease onset, course of disease, number of hospitalizations, and level of education, was collected from each participant.

### Variables and Instruments

#### Schizophrenia Quality of Life Scale

SQLS is a developed, disease-specific scale, widely used to assess the impact of schizophrenia on a patient's life, and has both internal reliability and structural validity (16). The Chinese version of the SQLS is translated by Luo et al. (17). SQLS contains 30 items that assess QoL from three factors, including psychosocial (15 items), motivation/energy (7 items), and symptoms/side effects (8 items). Each item ranges from 0 (never) to 4 (always). In addition, each factor ranges from 0 to 100, and a lower SQLS score represents a better QoL.

#### Self-Rating Scale of Systemic Family Dynamics

SSFD is the only scale to evaluate localized (Chinese) family dynamics, compiled and revised based on the Heidelberg family dynamic theory (18). The reliability and validity of SSFD were satisfactory (19). SSFD has a total of 30 items and each item is related on a five-point scale (1 = completely consistent to 5 = completely inconsistent). The SSFD evaluated the family dynamics of each participant from four dimensions, including family atmosphere (with lower scores reflecting better family communication), personalization (with lower scores reflecting higher self-discrimination ability), system logic (with lower scores reflecting more diverse ways of thinking), and disease concepts (with lower scores reflecting a greater belief in internal attribution and self-adjustment). Scales with lower scores represent better family dynamics. In brief, family atmosphere refers to the strong positive or negative emotional perception conveyed to the individual in the family environment; personalization is also called self-discrimination, and refers to the ability of an individual to separate emotion from reason; system logic refers to the ability to think about problems from different angles, meaning a flexible way of thinking; and disease concepts indicate the idea that diseases are caused by traditional beliefs, and that the individual can cope with the disease through internal adjustments and disregard external support.

#### Stigma Scale for Mental Illness

SSMI is a stigma assessment scale widely suitable for patients with mental illness in China, and its internal consistency coefficient alpha is 0.90 (20). The 32-item SSMI scale is used to assess the self-stigma in schizophrenia patients *via* three concepts, including social (14 items), functioning (8 items), and treatment (10 items), rated from 0 (never) to 3 (always). A higher score means stronger stigma.

In addition, 10 factors investigated in the SQLS, SSFD, and SSMI scale were considered as observed variables. The QoL, family dynamics, and stigma were regarded as latent variables.

### Statistical Analysis

The demographic data of the patients from the community and hospital were compared by a chi-square test. The factor scores were calculated according to the scoring principle of the scale. SPSS17.0 (SPSS Inc., Chicago, IL, USA) and AMOS24 (IBM Corporation, Armonk, NY, USA) were used for descriptive, correlation, and regression analyses. The correlations between observed variables were evaluated by Spearman's rank correlation analysis, and then the White's test was used to perform heteroskedasticity. A two-sided *p-*value <0.05 was considered statistically significant.

Structural equation modeling (SEM) was used to test the relationships between the three latent variables (QoL, stigma, and family dynamics) and generated the mediation model. Model fit was evaluated using a chi-square test (χ^2^/df), root mean square error of approximation (RMSEA), comparative fit index (CFI), incremental fit index (IFI), and Tucker–Lewis index (TLI). In brief, χ^2^/df > 2 (21), RMSEA values <0.1 (22), and CFI, IFI, as well as TLI values > 0.90 (23) implied good model fit. The fit indices should be interpreted collectively. Standardized coefficients (β) were examined to conduct regression path analyses. Mediation was analyzed using bootstrapping, and an indirect effect was considered significant when it did not contain 0 at the 95% interval. Furthermore, demographic factors (including age, age of onset, course of disease, and education level) that affected the independent variables, intermediate variables, and dependent variables were determined as covariates. Then, we controlled for covariate effects in the mediation model. If the independent variable did not affect the dependent variable upon addition of the mediator to the model, full mediation was established.

## Results

### General Demographics

Of the 600 sets of scales collected, 447 sets were valid, including data from 281 community patients and 166 inpatients. Demographic data are presented in Table 1. There was no significant difference between community patients and inpatients in age of onset, course of disease, and level of education (all *p* > 0.05). The inpatients were significantly older than community patients (*p* < 0.001).

**Table 1:** Demographics and disease characteristics of patients (*N* = 447).

	**Inpatients** **(*n* = 166)**	**Community patients** **(*n* = 281)**	***p***
	**M ± SD**	**M ± SD**	
Age	45.52 ± 9.57	39.25 ± 15.90	0.000^***^
Age of disease onset	27.38 ± 8.07	27.20 ± 8.44	0.096
Course of disease	18.45 ± 10.01	18.38 ± 9.54	0.113
Gender	1.42 ± 0.49	1.48 ± 0.50	0.162
Education level	2.52 ± 0.78	2.77 ± 0.82	0.902

*Gender and education levels are dummy variables. Male = 1, female = 2, primary school education = 1, junior high school education = 2, high school degree = 3, bachelor's degree = 4, master's degree and above = 5*.

*** *p < 0.001*.

### Correlation Analysis of Factors in the Three Scales (SQLS, SSFD, and SSMI)

Prior to mediation regression, bivariate Spearman's correlations were used to explore the relationships between family atmosphere, QoL, and stigma variables (see Table 2). In brief, good family atmosphere and higher personalization (low score) was significantly associated with worse system logic (high score, *p* < 0.01), better disease concepts (*p* < 0.01), lower QoL (high score on psychosocial, motivation and energy, and symptoms and side effects; all *p* < 0.01), as well as lower stigma (low score on social, competence, and treatment; all *p* < 0.01).

**Table 2:** Correlation analysis for 10 factors in family dynamics, quality of life, and stigma.

**Variables**	**Observed items**	**1**	**2**	**3**	**4**	**5**	**6**	**7**	**8**	**9**	**10**
Family dynamics	1 Family atmosphere	1									
	2 Personalization	0.663^**^	1								
	3 System logic	−0.341^**^	−0.392^**^	1							
	4 Disease concepts	0.526^**^	0.529^**^	−0.433^**^	1						
Quality of life	5 Psychosocial	−0.334^**^	−0.273^**^	0.109	−0.157^**^	1					
	6 Motivation/energy	−0.455^**^	−0.409^**^	0.149^**^	−0.263^**^	0.398^**^	1				
	7 Symptoms/side effects	−0.246^**^	−0.237^**^	0.08	−0.143^**^	0.674^**^	0.323^**^	1			
Stigma	8 Social	0.451^**^	0.509^**^	−0.176^**^	0.293^**^	−0.453^**^	−0.427^**^	−0.289^**^	1		
	9 Competence	0.476^**^	0.530^**^	−0.157^**^	0.238^**^	−0.372^**^	−0.491^**^	−0.250^**^	0.760^**^	1	
	10 Treatment	0.404^**^	0.466^**^	−0.108	0.276^**^	−0.350^**^	−0.388^**^	−0.288^**^	0.665^**^	0.625^**^	1

** *p < 0.01*.

Moreover, results revealed that better QoL (low score on psychosocial, motivation and energy, and symptoms and side effects) connected with a higher score on social, competence, and treatment (all *p* < 0.01), which represented higher stigma.

### Mediation Analysis of Subjective QoL Between Family Dynamics and Stigma

**Figure 1:**
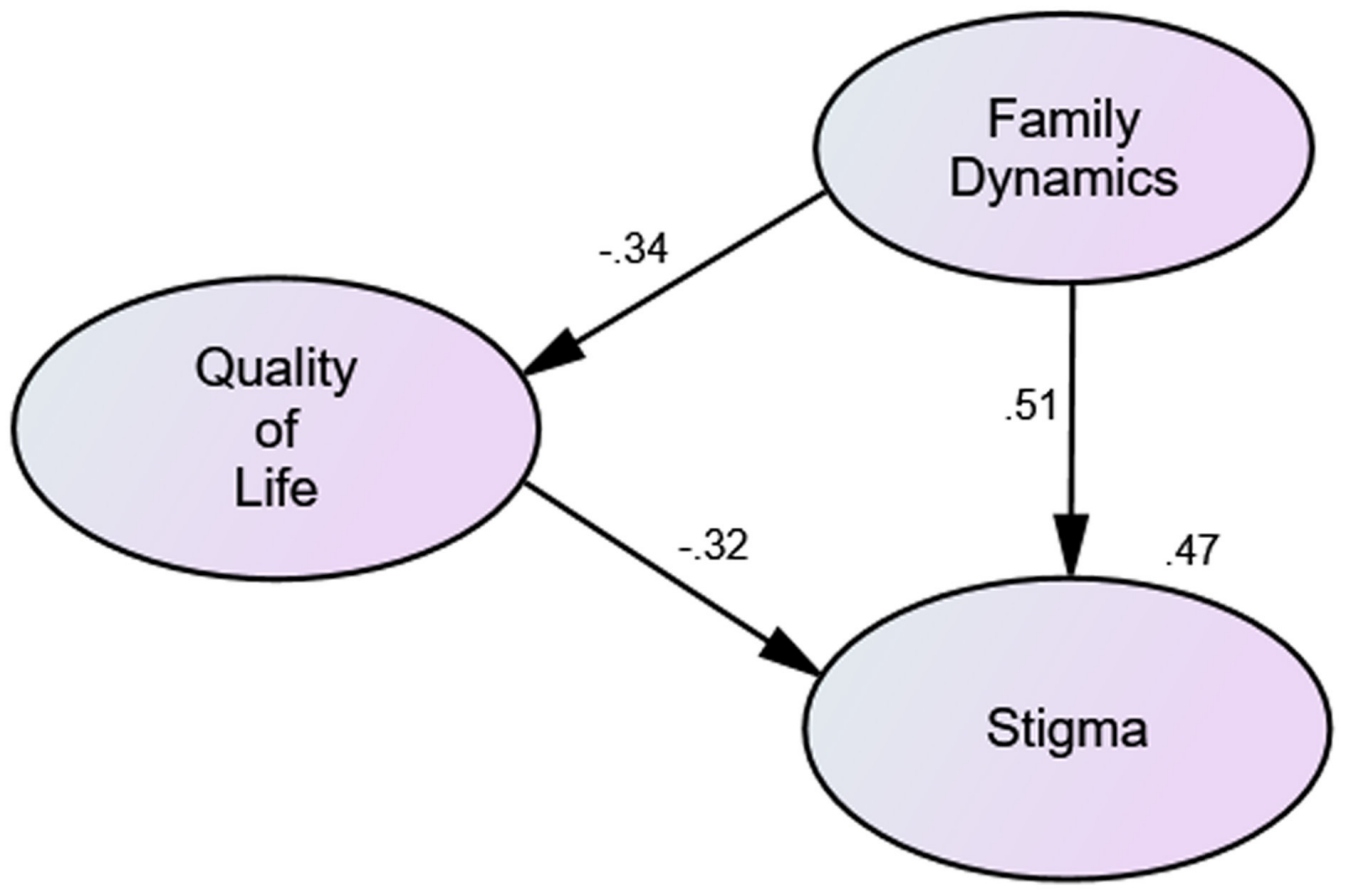
Mediating effects of subjective quality of life on the relationship between family dynamics and stigma.

Based on the above results, we hypothesized that QoL acted as a mediating variable between family dynamics and stigma. To validate this hypothesis, SEM was constructed to predict the relationships among these three variables. Results indicated that the overall model fit was acceptable (χ^2^/df = 3.401, RMSEA = 0.073, CFI = 0.979, IFI = 0.98, and TLI = 0.964). As illustrated in Figure 1 and Table 3, family dynamics negatively predicted QoL (β = −0.34, *p* < 0.001), and thus good family dynamics (low score) was significantly associated with worse QoL (high score). QoL negatively predicted stigma (β = −0.32, *p* < 0.001); that is, individuals with better QoL (low score) were more likely to have a higher level of stigma (high score). However, hypothesis 1 was not supported. In addition, family dynamics could directly predict stigma (β = 0.51, *p* < 0.001). Specifically, good family dynamics (low score) was connected to a lower level of stigma (low score). Hence, hypothesis 2 was supported.

**Table 3:** Path analysis of family dynamics, quality of life and stigma.

**Path**	**Unstandardized coefficient**	**β**	**SE**	**CR**	***p***
Family dynamics → quality of life	−1.816	−0.341	0.216	−8.398	^***^
Family dynamics → stigma	0.503	0.505	0.057	8.854	^***^
Quality of life → stigma	−0.073	−0.319	0.011	−6.685	^***^

*SE, standard error; CR, critical ratio*.

*** *p < 0.001*.

Next, the mediation analysis revealed that QoL was found to be a significant mediator between family dynamics and stigma, which supported hypothesis 3. Due to these regressions being significant, the bias-corrected bootstrap method was used to examine the indirect effect of QoL. As illustrated in Table 4, the indirect effect of subjective QoL on family dynamics through stigma was 0.109, and the 95% bootstrap CI for the total effect of the model did not contain a zero value [95% CI (0.091, 0.186), *p* < 0.001]. The direct effect of family dynamics on the stigma index did not include a zero value [*B* = 0.505, 95% CI (0.400, 0.625), *p* < 0.001]. This further indicated that QoL had a significant mediating effect on the relationship between family dynamics and stigma. The effect size of the mediating effect was 17.75%.

**Table 4:** Mediation analysis on family dynamics, quality of life and stigma.

**Path**	**SE**	**Coefficient**	**Bias-corrected 95% CI**			**Percentile 95% CI**		
			**Lower**	**Upper**	***p***	**Lower**	**Upper**	***p***
Family dynamics → Quality of life → Stigma	0.024	0.109	0.091	0.186	0.000	0.089	0.184	0.000^***^
Family dynamics → Stigma	0.057	0.505	0.400	0.625	0.000	0.392	0.616	0.000^***^

*SE, standard error; CI, confidence interval*.

*** *p < 0.001*.

## Discussion

Schizophrenia is a complex mental disease affected by both internal and external factors (24). Family, behavior, and perceived QoL are considered as critical factors influencing disease status. Hence, we analyzed the correlations between QoL, family dynamics, and stigma using three scale instruments. Moreover, we investigated whether the QoL mediated the relationship between family dynamics and stigma in individuals with schizophrenia. Results indicated that both family dynamics and QoL could predict stigma. In brief, individuals with better QoL were more likely to have a higher level of stigma, and good family dynamics was connected with a lower level of stigma. We also observed that good family dynamics was significantly associated with worse QoL. Importantly, QoL played a significant mediating role in the relationship between family dynamics and stigma. Even when the covariates were controlled, all pathways in these models were still significant.

In the present study, these three scales (SQLS, SSFD, and SSMI) included 10 observed variables. Correlation analysis indicated that most variables were significantly correlated with each other, indicating potential relationships between the variables included in the three scales. We found that both family dynamics and QoL had predictive effects on patients' stigma. Hesse et al. (25) indicated that broader interventions to improve the family atmosphere for patients and their caregivers may also improve negative self-concepts and paranoia. In addition, we found that the higher personalization (low score) was observably connected with a low level of stigma. This result might be attributed to the fact that individuals with a higher degree of personalization had better emotional control, and thus felt less stigma. There was strong evidence to support the use of family intervention, and that educating all patients and their families regarding the nature of the disease was effective in treating schizophrenia patients (26). These studies further indicated the potential role of family dynamics in reducing stigma in patients. Ohi et al. (27) indicated that the impaired physical activity of patients with schizophrenia was affected by psychosocial and motivation/energy subscales. Similarly, we also found these potential relationships. Overall, the significant connections between these observed variables further revealed the complex relationships between the three latent variables. However, few studies have reported the complex relationship of these 10 variables in schizophrenia patients. Therefore, the specific mechanism needs follow-up research.

We observed a direct predictive effect among three indicators. For hypothesis 1, we explored the relationship between QoL and stigma. Surprisingly, results revealed that patients' QoL negatively predicted stigma; that is, individuals with better QoL were more likely to have a higher level of stigma. This was inconsistent with the results of previous studies. Numerous studies have shown that better QoL is usually accompanied by lower stigma (9). QoL includes different dimensions, such as physical and mental health, psychological and social well-being, and the ability to carry out the activities of daily life (28). In recent years, the majority of studies on QoL have focused on white populations. Preliminary evidence has claimed that QoL is affected by different sociocultural and cultural factors. Thus, the results of these Western studies may not be valid in Chinese societies due to their different socio-cultural backgrounds (29). In the Chinese context, the special manifestation of stigma associated with schizophrenia was shaped by cultural connotations of Confucianism, the centrality of “face,” and the contemptuous etiological beliefs regarding mental illness (30) that influenced the higher stigma of Chinese individuals toward schizophrenia patients. Combined with our findings and Chinese sociocultural contexts, patients usually return to their family to recover after completing the acute treatment for schizophrenia in hospital. However, the chronic features of mental disorders create an increased burden of care for family members (31). It is easy to imagine that while meeting the care needs of patients and improving their QoL, it will also put more pressure on patients to be taken care of, as well as higher stigma. In addition, in order to achieve good QoL, patients may adopt a defensive attitude to deal with cognitive conflicts caused by differences in social and family environments after discharge. However, it is this defense mechanism that may generate a stronger sense of stigma. The above may explain why we obtained different results from previous reports. Moreover, the test scales were filled out by the participants themselves, which were subjective and random. Thus, it is needed to include larger subject cohorts to verify our findings.

Results indicated that family dynamics was positively associated with stigma—that is, better family dynamics (low score) was connected with a lower level of stigma, which supported our hypothesis 2. Evidence has highlighted that family interventions have always played an essential role in the treatment of schizophrenia (32). Numerous studies conducted in different countries have indicated that the family of individuals with schizophrenia demonstrated high levels of criticism, hostility, or overinvolvement, being defined as high expressed emotion (EE) (33). Meanwhile, Masako and Solomon (34) found that patients living with families with high EE experienced higher levels of stigma because they could be under an increased burden of care. Thus, preventing hostility, open criticism, and excessive emotional involvement with the patient's family members and creating a problem-solving family atmosphere could reduce the burden of schizophrenia on the patient and their relatives (35). In this study, better family dynamics mainly included good communication between family members and patients, respecting their personalization, encouraging diverse thinking, and making them believe in themselves. Consistent with our findings, it is generally assumed that flexible family values and good communication allow family members to express themselves freely and feel accepted, thereby reducing stigma. Furthermore, results indicated that good family dynamics was significantly associated with poor QoL. Generally, a higher QoL was positively correlated with good family interaction or cohesion (36). This finding was inconsistent with previous studies. Participants included in this study came from the community-based mental health service center or hospital. Most of them might have gone back to the community after being discharged from the service center or hospital, so they needed to manage their lives independently, such as coping with stigma from society, enduring residual symptoms and side effects, and insisting on treatment to reduce the rate of relapse and readmission (37). Evidence indicated that the psychosocial and motivation/energy of community patients significantly deteriorated after discharge (37). In addition, the relapse rate in the first year of discharge was as high as 52%. Depressive symptoms and poor adherence were significant relapse predictors (38). Thus, when patients were in a hospital and separated from real life, they could avoid thinking about life and social issues, but after being discharged, they had to face the transition to the community and could feel out of control and anxious about the changes in their living environment. Conceivably, patients living with good family dynamics could receive adequate support and understanding, but once they entered the community, patients experienced cognitive conflict due to the gap between society and family. The evidence suggested that the better the family dynamics, the stronger the cognitive conflict, thus leading to a worse QoL.

Importantly, the structural pathway modeling further suggested that QoL played a crucial mediating role between family dynamics and stigma among the schizophrenia patients, which supported hypothesis 3. In brief, family dynamics could directly predict the degree of stigma and also exerted an indirect influence on stigma *via* the mediating effect of QoL. Specifically, we observed that good family dynamics could reduce the stigma toward patients through the mediating effect of reduced QoL. In a study reporting stigma related to children with epilepsy and their parents, the authors found that when family cohesion levels were higher, both children and parents reported lower levels of stigma and better QoL (36), thus partly inconsistent with our findings. This difference may also be caused by different cultural backgrounds. The research study by Mendes et al. was based on Western culture. However, our research was conducted in China. Combined with the findings above, good family dynamics caused patients to experience strong cognitive conflicts that led to poor QoL. Poor QoL was associated with lower stigma and mediated the relationship between family dynamics and stigma. Thus, better family dynamics predicted lower stigma.

Several studies on reducing stigma experienced by schizophrenia patients have been reported. In terms of social–environmental interventions, although good family dynamics provide patients with enough care and love, social stigma leads to stigma in patients as well as their family members, which may make them fear social evaluation and cause social isolation (39). Previous studies indicated that the intervention of single factor has limited improvement in patients' stigma (40, 41). In this study, we found that QoL acted as a mediating variable between family dynamics and stigma. Thus, reducing the stigma of patients with schizophrenia requires the interaction of multiple factors, including the patient's own effort, family support, and a friendly social environment.

To the best of our knowledge, this is the first study to explore the mediating role of QoL between family dynamics and stigma. These findings suggested that the research on stigma experienced by schizophrenia patients should focus on the interaction of various factors in patients' lives. However, several limitations should be noted. First, our participants were from Pudong New Area of Shanghai, China. The sample ratio of inpatients and community patients was not balanced. This research may not be representative of individuals with acute schizophrenia. Second, cross-sectional studies are observational and cannot draw conclusions regarding causality. Finally, the focus of this article is to emphasize the patient's self-stigma, without considering the QoL and stigma of family members. Therefore, longitudinal research is needed in the future to explore the causal relationship between these three factors. We should also include more comprehensive factors and consider the feelings of family members.

The present study indicated that QoL had a mediating effect on the relationship between family dynamics and stigma. Factors influencing QoL and family dynamics should be considered comprehensively. These findings are of great value for translational treatments as they assist to better understand the factors influencing stigma. The complex association between factors and mediators suggests that integrated and personalized programs might be necessary. Specifically, interventions regarding QoL and family dynamics are required to reduce self-stigma of patients with schizophrenia.

## Data Availability Statement

The original contributions presented in the study are included in the article/supplementary material, further inquiries can be directed to the corresponding author/s.

## Ethics Statement

The studies involving human participants were reviewed and approved by the Ethics Committee of Shanghai Pudong Mental Health Center. The patients/participants provided their written informed consent to participate in this study.

## Author Contributions

LW and HQ: conception and design of the research. CH: acquisition of data. HQ: analysis and interpretation of data. YC: statistical analysis. HQ and LW: obtaining funding. LW and YC: drafting the manuscript. YC and HQ: revision of manuscript for important intellectual content. All authors have read and approved the final manuscript.

## Conflict of Interest

The authors declare that the research was conducted in the absence of any commercial or financial relationships that could be construed as a potential conflict of interest.

## Publisher's Note

All claims expressed in this article are solely those of the authors and do not necessarily represent those of their affiliated organizations, or those of the publisher, the editors and the reviewers. Any product that may be evaluated in this article, or claim that may be made by its manufacturer, is not guaranteed or endorsed by the publisher.
